# The GlnR Regulon in *Streptococcus mutans* Is Differentially Regulated by GlnR and PmrA

**DOI:** 10.1371/journal.pone.0159599

**Published:** 2016-07-25

**Authors:** Yi-Ywan M. Chen, Yueh-Ying Chen, Jui-Lung Hung, Pei-Min Chen, Jean-San Chia

**Affiliations:** 1 Department of Microbiology and Immunology, College of Medicine, Chang Gung University, Taoyuan, Taiwan; 2 Graduate Institute of Biomedical Sciences, College of Medicine, Chang Gung University, Taoyuan, Taiwan; 3 Molecular Infectious Disease Research Center, Chang Gung Memorial Hospital, Taoyuan, Taiwan; 4 Department and Graduate Institute of Microbiology, College of Medicine, National Taiwan University, Taipei, Taiwan; LSU Health Sciences Center School of Dentistry, UNITED STATES

## Abstract

GlnR-mediated repression of the GlnR regulon at acidic pH is required for optimal acid tolerance in *Streptococcus mutans*, the etiologic agent for dental caries. Unlike most streptococci, the GlnR regulon is also regulated by newly identified PmrA (SMUGS5_RS05810) at the transcriptional level in *S*. *mutans* GS5. Results from gel mobility shift assays confirmed that both GlnR and PmrA recognized the putative GlnR box in the promoter regions of the GlnR regulon genes. By using a chemostat culture system, we found that PmrA activated the expression of the GlnR regulon at pH 7, and that this activation was enhanced by excess glucose. Deletion of *pmrA* (strain ΔPmrA) reduced the survival rate of *S*. *mutans* GS5 at pH 3 moderately, whereas the GlnR mutant (strain ΔGlnR) exhibited an acid-sensitive phenotype in the acid killing experiments. Elevated biofilm formation in both ΔGlnR and ΔPmrA mutant strains is likely a result of indirect regulation of the GlnR regulon since GlnR and PmrA regulate the regulon differently. Taken together, it is suggested that activation of the GlnR regulon by PmrA at pH 7 ensures adequate biosynthesis of amino acid precursor, whereas repression by GlnR at acidic pH allows greater ATP generation for acid tolerance. The tight regulation of the GlnR regulon in response to pH provides an advantage for *S*. *mutans* to better survive in its primary niche, the oral cavity.

## Introduction

*Streptococcus mutans* is the principal etiologic agent of dental caries in humans [1]. The ability to quickly mount acid tolerance responses (ATR) upon exposure to sub-lethal acidic pH is a hallmark of its cariogenicity [2]. Among all ATR, the membrane-bound F-ATPase, which extrudes intracellular H^+^ at the expense of ATP [2, 3] is one of the major determinants to maintain cytoplasmic pH homeostasis. Additionally, the agmatine deiminase system, an analogue of the arginine deiminase system in *Streptococcus rattus* and *Streptococcus gordonii* [4], has been suggested to modulate intracellular pH by the production of ammonia and ATP [5]. Several lines of studies have shown that metabolism is closely linked to the stress responses in *S*. *mutans*. For instance, activation of the branched-chain amino acid (aa) biosynthetic pathway leads to reduction of lactic acid production and thus an acid resistance phenotype [6, 7]. Malolactic fermentation, which catalyzes the conversion of dicarboxylic L-malate to monocarboxylic lactic acid and CO_2_, enhances the capacity of *S*. *mutans* against lethal acidification [8, 9]. A recent study also indicates that metabolism of amino sugars in *S*. *mutans* leads to the production of ammonia and enhanced acid tolerance [10]. Our previous study demonstrates that the expression of the GlnR regulon is repressed by GlnR at pH 5.5 and that such regulation enhances the survival of *S*. *mutans* under acidic pH, presumably by reducing the synthesis of aa precursors and shifting the metabolism of citrate to pyruvate, with the consumption of H^+^ and potential ATP synthesis [11].

Nitrogen-dependent regulation in Gram-positive bacteria is governed mainly by GlnR (for Gln synthesis regulator) and TnrA (for *t**rans-*acting nitrogen regulation), although most species possess GlnR only [12]. The GlnR regulon is distributed widely in Gram positive bacteria. The expression of the GlnR regulon plays a central role in both nitrogen metabolism and cellular activities such as carbohydrate metabolism [13], virulence [14], and stress responses [11]. Genes encoding GlnR (*glnR*) and glutamine synthetase (GS) (*glnA*) are arranged as an operon in many of the low-G+C content Gram-positive bacteria, and TnrA is encoded as a monocistronic message. TnrA and GlnR in *Bacillus subtilis*, members of the MerR family of transcription factors, are highly conserved at the N-terminal helix-turn-helix (HTH) DNA binding domains, and both recognize a highly homologous operator sequence (5’-TGTNA-N_7_-TNACA), the GlnR Box [15–17]. Conversely, GlnR is activated by feedback-inhibited GS under nitrogen excess, whereas feedback-inhibited GS inhibits the DNA binding activity of TnrA [18–21]. Furthermore, GlnR acts as a repressor, while TnrA is a dual regulator that represses or activates various target genes [22]. For instance, TnrA activates the expression of its own and represses *glnRA* expression under nitrogen limitation, whereas GlnR represses *glnRA* and *tnrA* under nitrogen excess [23]. In addition to cross-regulation at the transcriptional level, TnrA is protected from proteolysis via a membrane-associated complex of AmtB and GlnK under nitrogen-limited growth in *B*. *subtilis* [24, 25]. The membrane association of TnrA also reduces its inhibitory effect on GS, under which condition the activity of GS is most needed [26].

We identified a GlnR regulon composed of 6 genes/operons in *S*. *mutans* GS5 by examination of the genome sequence of *S*. *mutans* UA159 [27] previously. The regulon includes the *citBZC* operon (encoding enzymes involved in the α-ketoglutarate and glutamate biosynthesis pathway), the *glnRA* operon, the SMU_806c-SMU_805c and *glnQHMP* operons (both encoding putative aa ABC transporters), SMU_807 (encoding a putative membrane protein), and the *nrgA-*SMU_1657c operon (encoding an ammonium transporter and a PII-like protein, respectively). GlnR represses the expression of the GlnR regulon at acidic pH to promote optimal acid tolerance [11]; however, the molecular basis of the regulation and whether additional regulatory proteins are involved in the pH-sensitive regulation were unclear. Our recent genomic analysis of *S*. *mutans* GS5 identified a putative MerR family regulator, SMUGS5_RS05810, which possesses a GlnR-like HTH domain. Here we demonstrated that SMUGS5_RS05810 also participates in the regulation of the GlnR regulon. Different from TnrA of *B*. *subtilis*, the activity of SMUGS5_RS05810 is mainly modulated by pH, and thus this locus is designated PmrA for pH-sensitive MerR family regulator A in this study.

## Materials and Methods

### Bacterial strains and growth conditions

The bacterial strains used in this study are listed in Table 1. *S*. *mutans* GS5 and its derivatives were cultivated routinely in Brain-Heart infusion (BHI, Difco) broth in 10% CO_2_ at 37°C. When necessary, erythromycin (Em) at 10 μg ml^-1^, kanamycin (Km) at 500 μg ml^-1^, and spectinomycin (Sp) at 500 μg ml^-1^ were included in the culture media. Recombinant *E*. *coli* strains were grown in LB broth supplemented with ampicillin (Ap) at 100 μg ml^-1^ and Km at 50 μg ml^-1^ as needed.

**Table 1:** Bacterial strains used in this study.

*S*. *mutans* strain	Relevant phenotype(s)^a^	Description	Source
GS5	GlnR^+^, PmrA^+^	Wild-type strain	H. K. Kuramitsu
ΔGlnR	Em^r^, GlnR^-^	GS5 *glnR*::*erm*	This study
ΔPmrA	Km^r^, PmrA^-^	GS5 *pmrA*::Ω*kan*	This study
ΔGlnR_PmrA	Km^r^, Em^r^, PmrA^-^, GlnR^-^	GS5 *glnR*::*erm*, *pmrA*::Ω*kan*	This study
GlnR+/ΔGlnR	Sp^r^, Em^s^, GlnR^+^	*glnR*::*erm* in ΔGlnR was replaced with an intact *glnR* gene tagged with *spe*	This study
PmrA+/ΔPmrA	Sp^r^, Km^s^, PmrA^+^	*pmrA*::Ω*kan* in ΔPmrA was replaced with an intact *pmrA* gene tagged with *spe*	This study

^a^ r, resistance; s, sensitive; +, positive; -, negative.

The continuous chemostat culture system was used to evaluate pH-dependent regulation in *S*. *mutans* GS5. Briefly, wild-type GS5 and the PmrA-deficient strain (ΔPmrA) were cultivated in a Biostat Bplus bioreactor (Sartorius Stedim Biotech) in TY medium (30 g tryptone and 5 g yeast extract liter^-1^) supplemented with 20 mM or 100 mM glucose at a dilution rate of 0.3 h^-1^. The cultures were maintained at pH 7.2 and 5.5 by the addition of 2 N KOH. All cultures were cultivated for at least 10 generations to reach the steady state.

The biofilm formation of *S*. *mutans* and its derivatives was assessed in BHI or biofilm medium (BM) [28] supplemented with 10 mM glucose (BMG).

### General genetic manipulations, RNA isolation and quantitative real-time PCR (qPCR)

Restriction endonuclease and DNA modifying enzymes were purchased from New England Biolabs (NEB). PCRs were carried out by using Vent (NEB) or Blend *Taq* DNA polymerase (Toyobo). Primers used in this study are listed in S1 Table.

Total cellular RNA was isolated from streptococcal strains and further purified by using an RNeasy minikit (Qiagen). First-strand complementary DNA (cDNA) was generated from 2 μg of total cellular RNA with random hexamer primers. qPCR was used to evaluate the expression level of genes in the GlnR regulon. The reactions were carried out by using a Kapa SYBR fast kit (Kapa Biosystems) and a 7500 Fast real-time PCR system (Applied Biosystem). All reactions were run in triplicate, and at least three samples were analyzed. A melting curve analysis was performed with all pairs of primers to ensure the reaction efficiency. To be noted, the reaction efficiency of primers for *glnA*, *glnQ*, *gdhA*, *citB*, *nrgA* and *glnP* is 98.9%, 100%, 92.4%, 102.6%, 98.1% and 91.4%, respectively. The data were analyzed by using 7500 software v2.0.5. The change in the quantification cycle (Δ*Cq*) of each sample was normalized with 16S RNA. The Δ*Cq* derived from wild-type GS5 grown at neutral pH was used as a reference. The relative quantity of each sample was calculated as the Δ*Cq* of the sample compared to the Δ*Cq* of the reference using the formula 2^-ΔΔ*Cq*^.

### Construction of recombinant S. mutans strains

Gene inactivation in *S*. *mutans* GS5 was carried out by using a ligation-PCR mutagenesis strategy [29], and the genotypes of all recombinant strains were confirmed by PCR with primers located outside the antibiotic insertion site. Briefly, DNA fragments containing the 5’ and 3’ flanking regions of *glnR* were amplified from *S*. *mutans* GS5 by PCR with the primer pairs glnRA_969F plus glnRA_2033R and glnRA_2389F plus glnRA_3403R, respectively. These two PCR products were digested with XhoI and SphI, respectively, followed by ligation with the coding sequence of an Em resistance gene (*erm*) at the compatible ends [30]. A ligation mixture containing all three fragments was prepared and introduced into GS5 by natural transformation [31] to inactivate *glnR* by allelic exchange. The resulting strain was designated ΔGlnR. A similar approach, by using primer pairs pmrA_3574F plus pmrA_4544R and pmrA_4827F plus pmrA_5860R, was used to replace *pmrA* with a Km resistance gene cassette (Ω*kan*) [32]. The resulting two PCR products were digested with BamHI and XhoI, respectively, prior to ligating with the Ω*kan* fragment. The resulting strain was designated ΔPmrA. The inactivated *glnR* locus was also established in strain ΔPmrA to generate a double knockout strain (ΔGlnR_PmrA).

An intact *glnR* gene was established in strain ΔGlnR to restore a GlnR^+^ phenotype (strain GlnR+/ΔGlnR). Briefly, a DNA fragment containing the 5’ flanking region of *glnR* and a fragment containing the coding sequence of *glnR* and its 3’ flanking region were amplified from *S*. *mutans* GS5 by PCR with the primer pair glnRA_969F plus glnR_C_1945R and the pair glnR_C_1946F plus glnRA_3403R, respectively. These two fragments were digested with SphI and PstI, respectively, and then ligated to the 5’ and 3’ end of the coding sequence of a Sp resistance gene (*spe*) [33] at the compatible ends. The ligation mixture was introduced into strain ΔGlnR to replace the inactivated *glnR* with a *spe-*tagged *glnR* gene. The *spe* coding sequence is located at the 5’ end of *glnR* in the resulting strain, GlnR+/ΔGlnR. A similar approach was used to replace the inactivated *pmrA* with a *spe-*tagged *pmrA* gene in strain ΔPmrA. The primer pair pmrA_3574F plus pmrA_C_4860R and the pair pmrA_C_4861F plus pmrA_5860R were used to amplify a DNA fragment containing the intact *pmrA* gene, including both the native promoter and terminator sequences, and a DNA fragment containing sequences 3’ to *pmrA* from wild-type GS5, respectively, for ligation mutagenesis. These two PCR products were digested with SphI and PstI, respectively, prior to ligating with a *spe* gene at the compatible ends. The resulting strain, PmrA+/ΔPmrA, harbors an intact *pmrA* tagged with *spe* at its 3’ end.

### Growth kinetics

The growth kinetics of wild-type GS5 and the mutant strains in BHI was monitored using a Bioscreen C Microbiology Reader (Oy Growth Curve AB Ltd.) as previously described [34]. Briefly, overnight cultures of all strains in BHI were diluted at 1:20 in fresh BHI and grown to O.D._600_ = 0.3. The cultures were then diluted at 1:60 and inoculated in 300 μl fresh BHI in a 96-well microtiter plate. All samples were done in triplicate.

### Purification of recombinant GlnR and PmrA proteins

The coding region of *glnR* and *pmrA* were amplified from GS5 by PCR with primers MBP_glnR_F plus MBP_glnR_R and MBP_pmrA_F plus MBP_pmrA_R, respectively. The PCR products containing the coding sequence of *glnR* and *pmrA* were double-digested with EcoR+PstI and BamHI+PstI, respectively. The digested fragments were cloned in pMAL-c2X (NEB) at the compatible ends to generate constructs for purifying maltose binding protein (MBP)-tagged GlnR (MBP-GlnR) and PmrA (MBP-PmrA). The identity of the recombinant plasmids was confirmed by sequence analysis. The induction and purification under native condition were performed according to manufacturer’s recommendations. The identity of the purified protein was further confirmed by Matrix-Assisted Laser Desorption/Ionization Time of Flight Mass Spectrometry (MALDI-TOF).

### Electrophoretic mobility shift assay (EMSA)

Two 40-mer complementary primers containing the complementary sequence of the putative GlnR box and flanking regions in the promoter of the GlnR regulon gene were annealed and end-labeled with biotin by using a Biotin 3’ end DNA labeling kit (Pierce). The labelled probes were used in EMSA with purified recombinant GlnR and PmrA proteins. The reaction was carried out in a buffer containing 5 mM MgCl_2_, 20 mM Tris (pH 8.0), 2 mM DTT, 100 mM KCl, 1 μg BSA, and 0.5 μg salmon sperm DNA (Bioman). A 40-bp internal fragment of *citC* in 10-fold excess was used as a non-specific competitor. The purified MBP-GlnR and MBP-PmrA were mixed with 0.025 pmol of the probe in a final reaction volume of 20 μl. Reactions were carried out at 4°C for 30 mins. At the end of incubation, the reaction mixture was separated on a 6% polyacrylamide gel. The DNA and protein complexes were electrotransferred on to nylon membranes, and detected by using a chemiluminescent nucleic acid detection module kit (Pierce).

### Acid killing assay

The survival rate of wild-type *S*. *mutans* GS5 and the mutant strains at pH 3 was determined as previously described [11]. The viable counts of the culture at 30, 45, or 60 mins were determined by serial dilution and plating. The survival rate was calculated as a percentage of the viable cells at each time point compared to the number of viable cells prior to acid treatment. For each strain, at least three independent experiments were performed and all plating was done in triplicate.

### Biofilm assay

The biofilm formation of wild-type *S*. *mutans* GS5 and its derivatives was evaluated as previously described [35]. Briefly, overnight cultures in BMG were diluted at 1:100 in the same medium. An aliquot of 200 μl bacterial suspension was inoculated in a flat-bottom 96-well microtiter plate. All samples were done in quadruplicate. Plates were incubated at 37°C in 10% CO_2_. At each time point, the biofilm was stained with crystal violet and quantified by measuring the absorbance at 562 nm with a Micro-plate Reader (SoftMax^®^ Pro, Molecular Devices).

### The flow cell biofilm system

Overnight cultures of the wild-type and mutant strains in BHI were diluted in fresh BHI to an O.D._600_ of 0.01. An aliquot of 250 μl of the bacterial suspension was injected into the growth chamber of the continuous flow cell system (Stovall Life Sciences) with a 26G needle. The growth chamber was incubated at 37°C in an upside down position without medium flow for 4 h to allow for the adherence of the bacteria. The medium flow rate was then set at 5 ml^-1^ h^-1^ channel^-1^, and the incubation continued for additional 16 h. The biofilm in the flow chamber was stained with the SYTO 9/propidium iodide (PI) fluorochrome reagents (FilmTracer LIVE/DEAD biofilm viability kit, Invitrogen), and observed by confocal laser scanning microscope (CLSM).

## Results

### Identification and sequence analysis of SMUGS5_RS05810 (PmrA)

**Fig 1:**
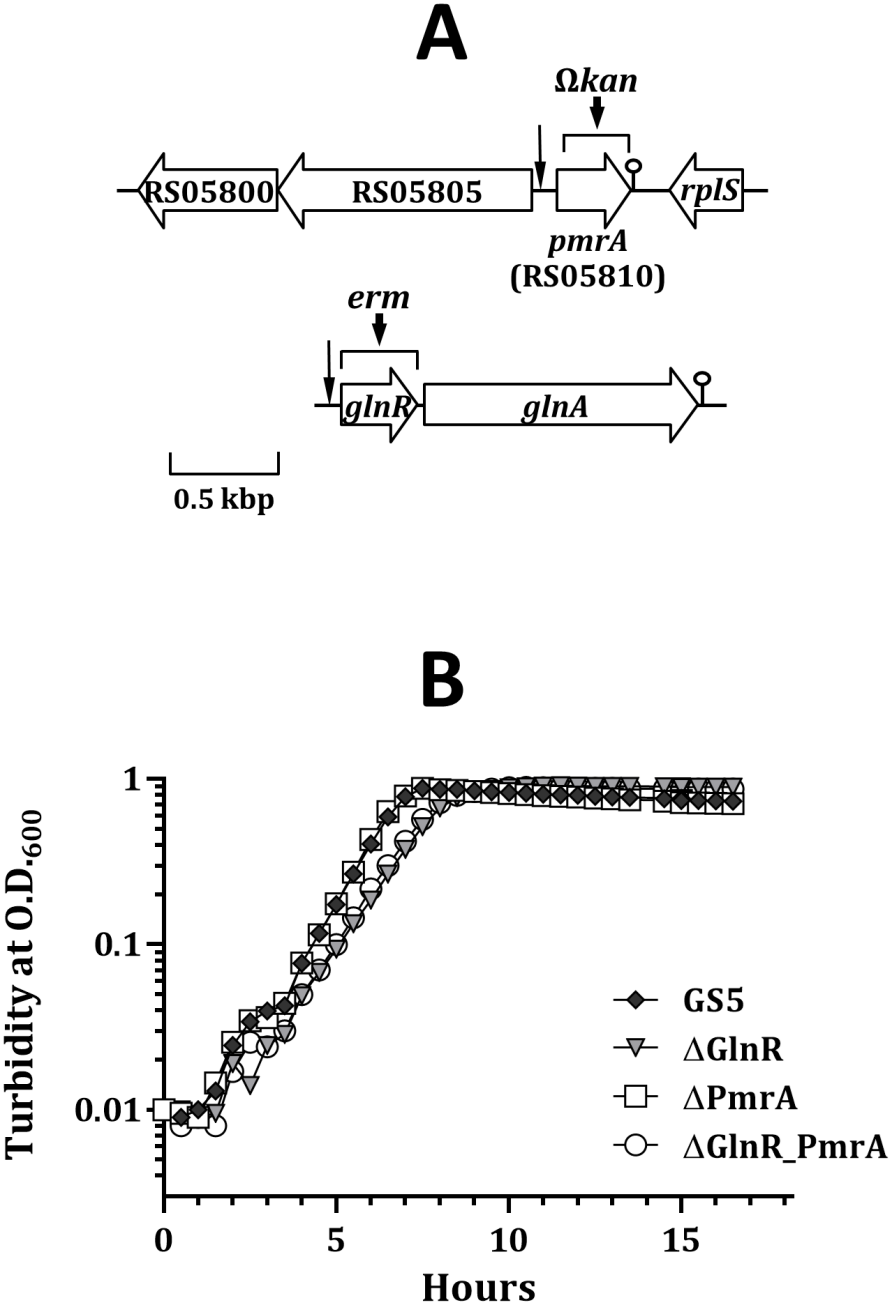
Schematic diagram of *S*. *mutans* GS5 SMUGS5_RS05810 (*pmrA*) and *glnRA*, and the growth kinetics of GS5 and its derivatives. (A) The relative locations of the predicated GlnR box in the 5’ flanking region of *pmrA* and *glnR* are indicated by a vertical arrow. The putative terminators of *pmrA* and *glnRA* are indicated by a lollypop-shaped symbol. The regions within *pmrA* and *glnR* that are replaced by Ω*kan* in strain ΔPmrA and *erm* in strain ΔGlnR, respectively, are indicated by a horizontal bracket. (B) The growth kinetics of wild-type *S*. *mutans* GS5, ΔGlnR, ΔPmrA, and the double knockout strain (ΔGlnR_PmrA) in BHI are shown. A representative graph of at least three experiments is shown.

**Fig 2:**
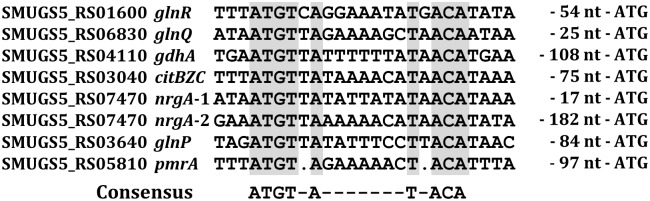
Alignment of the GlnR box of the *S*. *mutans* GS5 GlnR regulon genes. The distance between the GlnR box and the translational start site of each gene is listed.

In an attempt to investigate whether the GlnR regulon of *S*. *mutans* is regulated by a TnrA-like regulator, as seen in *B*. *subtilis*, the genome of *S*. *mutans* GS5 (NC_018089) was subject to various sequence analyses to search for MerR family regulators that harbor a GlnR-like domain. A locus, SMUGS5_RS05810, was then identified (Fig 1A). SMUGS5_RS05810, encoding a protein of 116 aa, was preceded by a putative ribosomal binding site. A putative rho-independent terminator (ΔG° = -13.40 kcal mol^-1^) was detected 1 base 3’ to the stop codon. A GlnR-like box was located 101 bases 5’ to the translation start site of SMUGS5_RS05810 (Fig 2). Only 33% identity (61% positive) at the deduced aa level was observed between SMUGS5_RS05810 and GlnR of *S*. *mutans* GS5, although both proteins possess a GlnR-like domain. Blast analysis revealed that SMUGS5_RS05810 is highly conserved among strains of *S*. *mutans* with known genome sequences. This locus is also found in the genomes of several streptococcal species of the mutans group, including *Streptococcus criceti* (WP_004229175), *Streptococcus macacae* (WP_003080257), *Streptococcus downei* (WP_002962070), and *Streptococcus sobrinus* (WP_019783562). Interestingly, homologs of SMUGS5_RS05810 are not found in the genomes of other viridans streptococci.

SMUGS5_RS05810 was designated PmrA based on the results of functional analysis (see below). For the continuity of the manuscript, PmrA is used in the remaining manuscript.

### Both GlnR and PmrA bind to the cognate GlnR box near the promoters of the GlnR regulon genes

**Fig 3:**
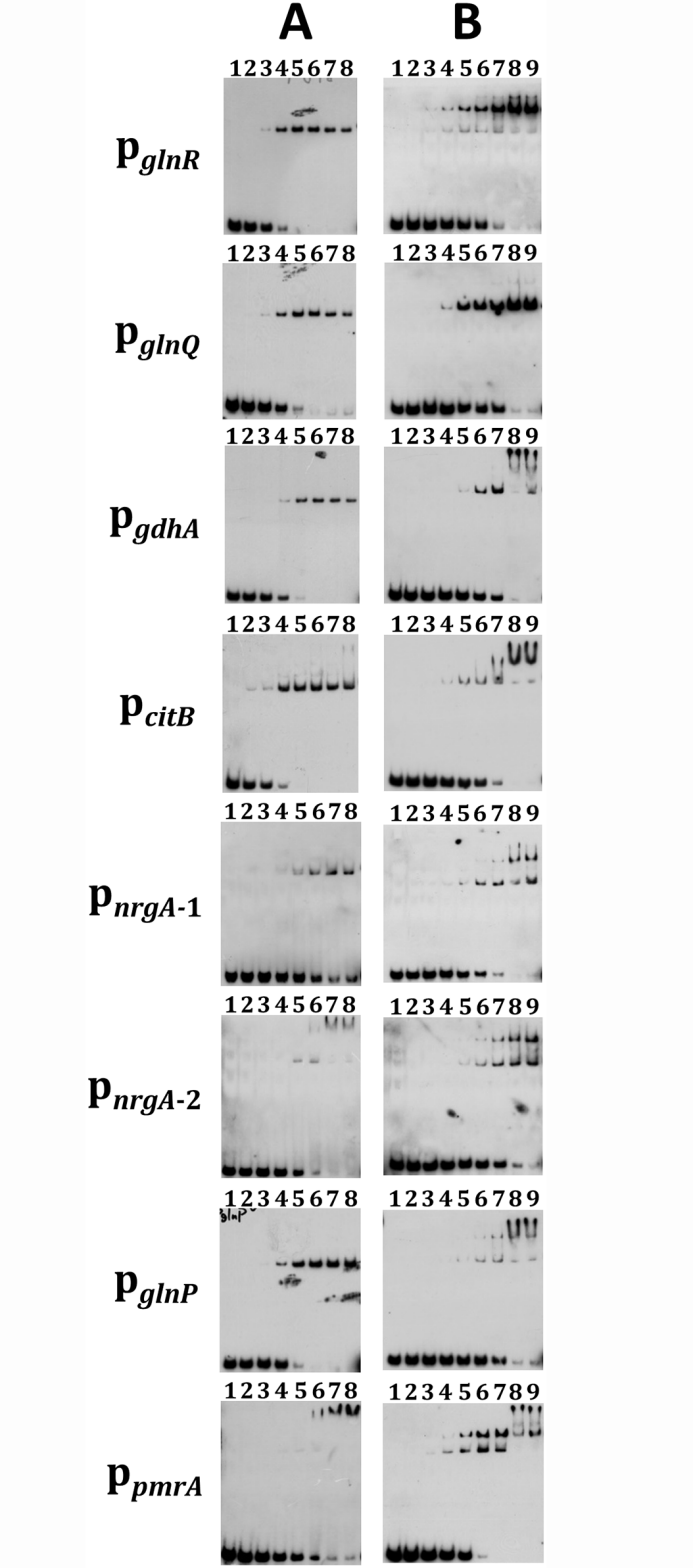
EMSA demonstrating the interaction of GlnR and PmrA to the promoters of genes in the GlnR regulon. The p_*glnR*_*-*, p_*glnQ*_*-*, p_*gdhA*_*-*, p_*citB*_*-*, p_*nrgA*-1_*-*, p_*nrgA*-2_*-*, p_*glnP*_*-* and p_*pmrA*_*-*specific DNA probes were mixed with various amounts of protein extracts. All reactions were performed with 0.025 pmol of biotin-labeled probes. (A) Lane 1, probe only. Lanes 2 to 7, use of 8 to 256 nM of MBP-GlnR in the reactions in twofold increments, respectively. Lane 8, use of 256 nM of MBP-GlnR with a non-specific competitor. In the reactions containing the p_*pmrA*_ probe, 16 nM to 512 nM of MBP-GlnR was used instead. (B) Lane 1, probe only. Lanes 2 to 8, use of 8 nM to 512 nM of MBP-PmrA in the reactions in twofold increments, respectively. Lane 9, use of 512 nM of MBP-PmrA with a non-specific competitor.

As only limited homology was observed between PmrA and GlnR in *S*. *mutans* GS5, whether PmrA could recognize GlnR box was investigated by EMSA. Among the open reading frames (ORFs) in the proposed GlnR regulon, the homolog of *S*. *mutans* UA159 SMU_807 was annotated as a pseudogene in the newly completed *S*. *mutans* GS5 genome. An examination of the GS5 genome revealed a single base deletion within the corresponding region which led to a truncated protein product of 66 aa, and thus the regulation of this truncated ORF was not pursued. Furthermore, recent sequence analysis revealed a GlnR box that is located 112 bases 5’ to the translation start site of *gdhA* (SMUGS5_RS04110, a NADPH-specific glutamate dehydrogenase) (Fig 2), thus *gdhA* is included in the following studies. An alignment of the putative GlnR boxes of all genes analyzed in this study is shown in Fig 2. To be noted, two potential GlnR boxes were found in the promoter of *nrgA* (p_*nrgA*_), 21 (*nrgA*-1) and 186 bases (*nrgA*-2) 5’ to the ATG of *nrgA*, respectively. The results of EMSA demonstrated that both GlnR and PmrA interacted with the putative GlnR box in the promoter regions of the GlnR regulon genes in *S*. *mutans* GS5 (Fig 3A and 3B). When the putative GlnR box sequence in the *glnR* promoter (p_*glnR*_) was mutated to interrupt the palindromic structure but maintain a wild-type GC content, both recombinant proteins failed to make a shift, indicating that both proteins recognized the GlnR box specifically (S1 Fig). A similar result was also observed with probes containing mutated GlnR box sequence in the promoters of *glnQ* (p_*glnQ*_) and *gdhA* (p_*gdhA*_) (S1 Fig), further confirming the binding specificity of GlnR and PmrA to the GlnR box. To rule out the possibility that GlnR and PmrA may interact with any intergenic DNA sequences under the experimental condition, a 40-bp DNA fragment that contains the putative promoter sequence of *dnaE* was used as a negative control. No shift was detected with both recombinant proteins (data not shown). Neither an internal fragment of *citB* nor the purified maltose binding protein was able to obtain a positive shift (data not shown). All above analyses confirmed the binding specificity of both proteins. Furthermore, with the exception of the promoter of PmrA (p_*pmrA*_), a lesser amount of GlnR, compared to PmrA, was required to have positive shifts with all other promoter regions, suggesting that GlnR was the dominant regulator of these genes. Of the two GlnR boxes 5’ to p_*nrgA*_, the one closest to the ATG of *nrgA* (p_*nrgA*-1_) was the major target for both GlnR and PmrA. It is noticed that multiple shift bands were observed with PmrA in all probes, and to a lesser degree, also with GlnR. As all probes used in EMSA contain only one GlnR box, this observation suggests that both recombinant proteins aggregate at high concentrations.

### The activity of PmrA is modulated by growth pH and carbohydrate concentrations

To evaluate the impact of GlnR and PmrA on the expression of genes in the GlnR regulon, strains ΔGlnR, ΔPmrA, and the double knockout mutant (strain ΔGlnR_PmrA) were constructed as detailed in Materials and Methods. The *glnR* gene was inactivated by the coding sequence of *erm* to maintain a wild-type level of the *glnA* expression, whereas the monocistronic *pmrA* was inactivated by a polar Ω*kan* cassette (Fig 1A). Strain ΔPmrA exhibited a growth rate similar to wild-type GS5 with a generation time of 42 min, whereas inactivation of *glnR* resulted in a generation time of 56 min in both ΔGlnR and ΔGlnR_PmrA mutant strains (Fig 1B). When the inactivated loci in strains ΔGlnR and ΔPmrA were replaced with intact *glnR* and *pmrA*, respectively, a wild-type growth rate was observed (data not shown).

**Fig 4:**
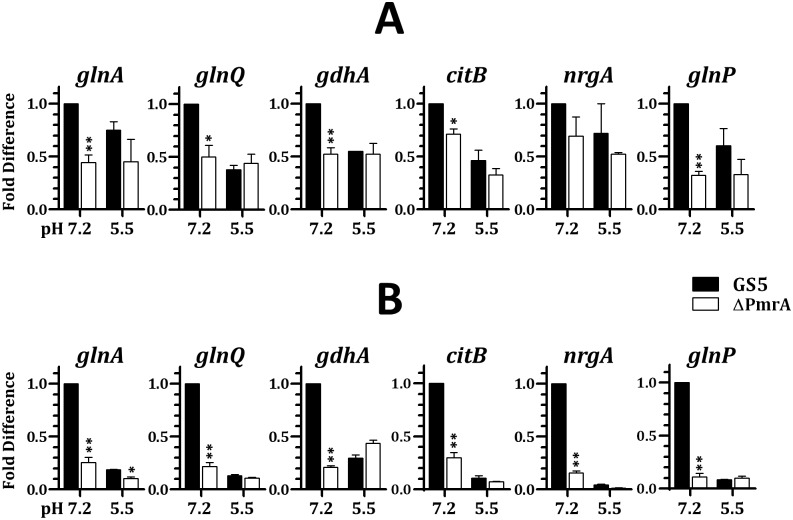
The impact of growth pH and carbohydrate concentrations on the regulatory activity of PmrA. Wild-type GS5 and strain ΔPmrA were grown in chemostat in TY containing 20 mM (A) or 100 mM glucose (B) at pH 7.2 or pH 5.5. The relative quantity of mRNA of the GlnR regulon gene was measured by qPCR. The Δ*Cq* of GS5 grown at pH 7.2 was used as the reference. Numbers are the means and standard deviations of three independent experiments. The significant difference between wild-type GS5 and strain ΔPmrA under each growth condition was determined by Student’s *t* test. **, *P* < 0.001; *, *P* < 0.01.

As the repression by GlnR under acidic growth pH is required for an optimal acid tolerance in *S*. *mutans* [11], the regulatory function of PmrA was examined in chemostat-grown cells at pH 7.2 and 5.5, in TY medium with 20 mM or 100 mM glucose by qPCR. To be noted, with most oral streptococcal species, when cells were cultivated in medium containing 20 mM glucose in a chemostat system, glucose is undetectable in the culture filtrates, i.e., glucose is the limiting growth factor. In the presence of 100 mM glucose, approximately 50 mM glucose could be detected in the culture filtrates, and thus glucose is in excess. In agreement with our previous observation [11], the expression of the GlnR regulon in wild-type GS5 was down regulated at pH 5.5 compared to pH 7.2 under both glucose concentrations (Fig 4A and 4B). This study also found that repression at pH 5.5 was more pronounced with 100 mM glucose (S2 Fig). To be noted, the expression level of all genes in the GlnR regulon in wild-type GS5 grown under 20 mM glucose was lower than that in cells grown under 100 mM glucose, regardless growth pH (S2 Fig), suggesting that the excess amount of nitrogen nutrients under 20 mM glucose promotes the repression of GlnR. Inactivation of *pmrA* led to down regulation of all genes examined at pH 7.2, and excess glucose further enhanced this regulation (Fig 4), indicating that PmrA acted as an activator mainly at neutral pH under glucose excess. At pH 5.5, no significant difference was observed between wild-type GS5 and strain ΔPmrA when cells were grown in the presence of 20 mM glucose (Fig 4A). When 100 mM glucose was included in the culture medium, significant activation by PmrA was only detected in *glnA*, indicating that PmrA remained active under this growth condition (Fig 4B). To confirm the specificity of regulation by PmrA, we measured the expression levels of *dnaA*, *dnaE*, and *gyrA*, genes without a putative GlnR box in the 5’ flanking region, in chemostat cultures of wild-type GS5 and strain ΔPmrA under different growth conditions. In all cases, a compatible expression level was detected in GS5 and strain ΔPmrA under all growth conditions used (S3 Fig), confirming that the regulation observed in Fig 4 was PmrA-specific. Taken together, PmrA, a pH-sensitive MerR family regulator, acts as an activator of the GlnR regulon mainly at pH 7.2, and this regulatory activity is augmented by an excess amount of glucose.

### The impact of PmrA regulation in the acid tolerance of *S*. *mutans*

**Fig 5:**
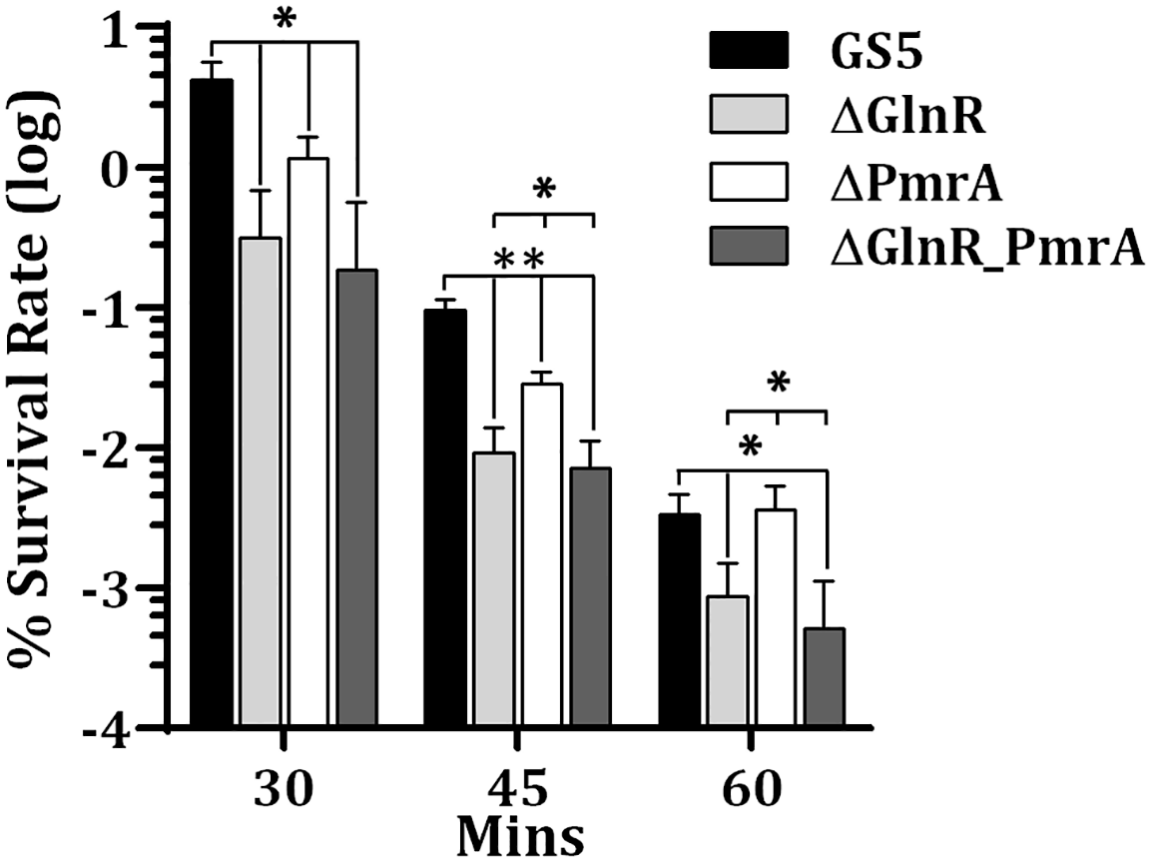
Acid killing assay demonstrating the impact of GlnR and PmrA on the viability of *S*. *mutans* at pH 3. The viable counts were determined by serial dilution and plating on BHI agar plates. Numbers are the mean and standard deviation of three independent experiments. Significant differences between strains were determined by Student’s *t* test. **, *P* < 0.005; *, *P* < 0.05.

As the expression of the GlnR regulon is essential for optimal acid tolerance in *S*. *mutans*, the role of PmrA in this process was evaluated in an acid killing assay (Fig 5). In agreement with previous observations [11], the survival rate of strain ΔGlnR was 78%, 90% and 74% lower than that of wild-type GS5 at 30, 45 and 60 min, respectively. The survival rates of the double knockout strain at all time points were similar to that of strain ΔGlnR. Inactivation of *pmrA* reduced the survival rate (70% lower than that of wild-type GS5) of *S*. *mutans* at pH 3 only in the initial 45 min of the killing experiment. The observed reduction may be the result of reduced amounts of GlnR in the *pmrA* knockout background compared to the wild-type host. It is also possible that additional targets regulated by PmrA contribute to the observed phenotype. Nevertheless, as PmrA was most active at neutral pH (Fig 4B), it was expected that the regulation of PmrA contributed moderately to acid tolerance.

### Both GlnR and PmrA regulate biofilm formation in *S*. *mutans*

**Fig 6:**
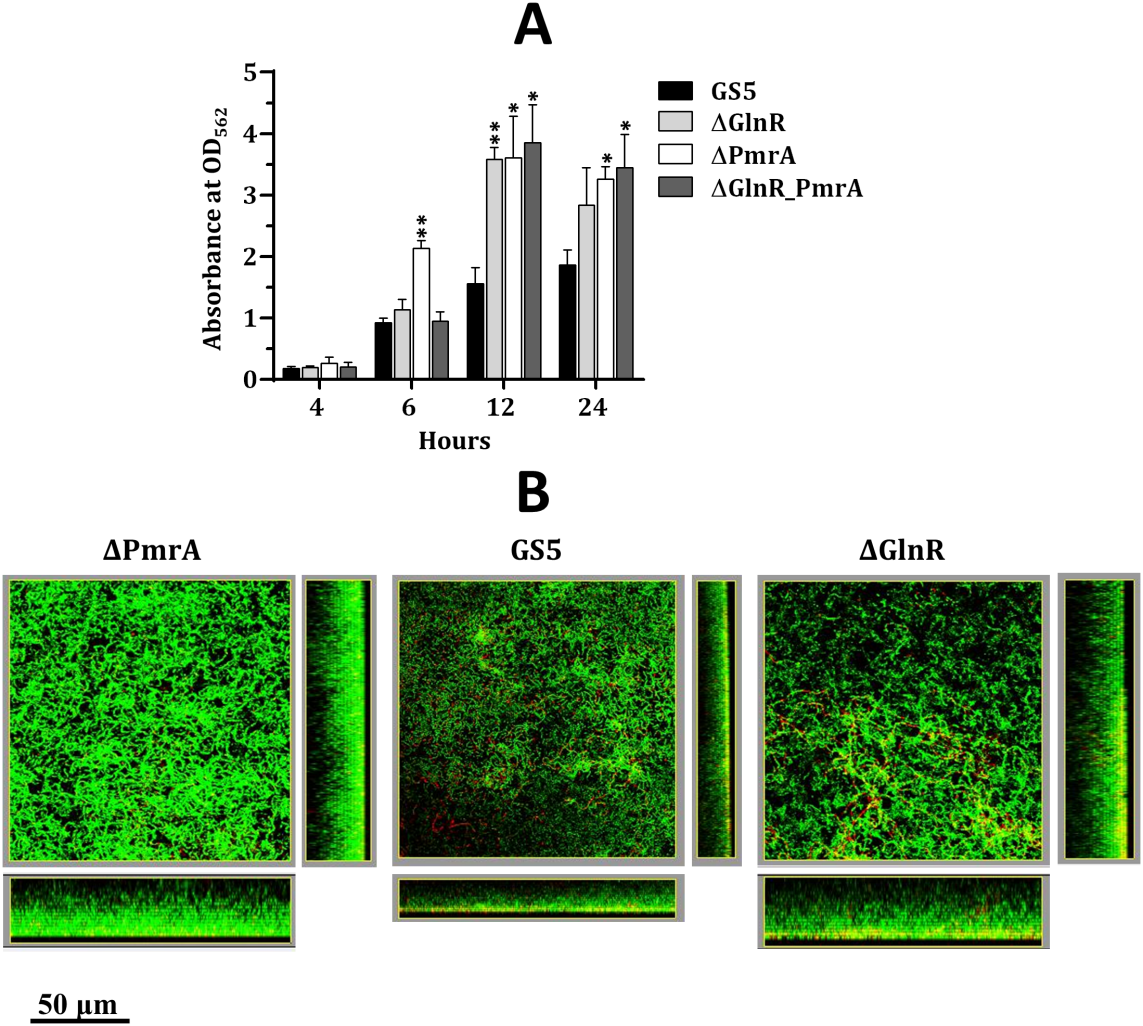
The biofilm formation of *S*. *mutans* GS5 and its derivatives. (A) The static biofilm formation. Cells were grown in BM containing 10 mM glucose. The amount of the crystal violet retained by the biofilm cells was measured by spectrophotometry. Values shown are the mean and standard deviation of three samples. Significant differences between GS5 and the mutant strains at each time point were determined by Student’s *t* test. **, *P* < 0.001; *, *P* < 0.01. (B) Structures of biofilms formed by wild-type *S*. *mutans* GS5 and its derivatives in the flow cell system. The biofilms were grown in BHI for 16 h. The biofilms were treated with the SYTO 9/PI fluorochrome reagents and visualized by CLSM. The live cells (green fluorescence) and dead cells (red fluorescence) are shown. Both the top view and side view of the biofilms are shown.

A recent study demonstrates that biofilm formation of *S*. *mutans* MT8148 is modulated by nitrogen nutrients [36]. Thus the possible impact of GlnR and PmrA on biofilm formation was evaluated. In static biofilm cultures, all strains showed time-dependent biofilm formation (Fig 6A). Interestingly, the biofilm mass of strain ΔPmrA was significantly greater than that of wild-type GS5 after 6 h cultivation, but the double knockout strain exhibited a wild-type level of biofilm formation. Since inactivation of *glnR* prolonged the doubling time (Fig 1B), reduced growth in the double knockout strain may outweigh the impact of PmrA on early biofilm formation. On the other hand, the elevated expression of the GlnR regulon in strain ΔGlnR did not impact early biofilm formation. At 12 and 24 h, all three mutant strains exhibited thicker biofilm than the wild-type strain did. As GlnR and PmrA represses and activates the GlnR regulon, respectively, this result suggested that enhanced colonization and/or cell-cell interaction are essential for the overall increase in the biofilm mass in strain ΔPmrA, whereas the expression of the GlnR regulon in the *glnR-*deficient background augmented biofilm formation.

To mimic the natural environment more closely and to determine the possible cause for enhanced biofilm formation in the mutant strains, the live and dead cells within the biofilms of wild-type GS5, ΔGlnR and ΔPmrA in a flow-cell system were examined. In agreement with the results of the static biofilm cultures, both mutant strains formed thicker biofilms (Fig 6B). The biofilms of wild-type GS5 and strain ΔPmrA concentrated the dead cells mainly in the bottom layer, whereas dead cells were distributed more evenly in the biofilm of strain ΔGlnR. As inactivation of *glnR* reduces the survival rate of *S*. *mutans* at acidic pH [11], it is suggested that the reduced acid tolerance in strain ΔGlnR is responsible for, at least in part, the cell death.

## Discussion

While most of streptococcal species utilize GlnR as the sole regulator for the GlnR regulon, this study demonstrated that *S*. *mutans* possesses a functional PmrA that activates the expression of the GlnR regulon mainly at neutral pH. In a search for transcriptional regulators of the MerR family, we identified 6 loci on the *S*. *mutans* GS5 genome. Among these loci, only GlnR and newly identified PmrA possess a GlnR-like DNA binding domain based on the sequence analysis and domain prediction. Thus, the expression of the GlnR regulon is likely to be controlled by GlnR and PmrA, but not other regulators in *S*. *mutans* GS5. The differential regulation of the GlnR regulon by PmrA and GlnR suggests that regulation by PmrA promotes optimal aa biosynthesis at neutral pH but contributes to acid tolerance moderately. Repression by GlnR at acidic pH shifts the metabolism from glutamine synthesis to ATP generation and proton extrusion, which directly enhances acid tolerance [11].

It is interesting that *pmrA* is present only in the cariogenic mutans streptococci, but not other oral streptococci. Since differential regulation by PmrA and GlnR directly links nitrogen metabolism and acid stress responses, the presence of PmrA could allow for full activation of GlnR regulon when aa biosynthesis is most needed for growth, and thus potentially provides additional advantages for cariogenic mutans streptococci. The expression of PmrA and GlnR and their regulatory function on the GlnR regulon in *S*. *mutans* are different from TnrA and GlnR in *B*. *subtilis*, as in *B*. *subtilis* the regulatory activity of both proteins is modulated by nitrogen nutrients [23]. Nitrogen availability is a frequent challenge for the soil bacterium *B*. *subtilis* and GlnR-mediated repression would tightly control the uptake of ammonia/amino-containing compounds and intracellular ammonia production for energy conservation [37]. However, pH alteration is a common and frequent insult for oral bacteria. Thus differences in the regulation mechanisms between these two species may reflect their natural niches.

It is well established that the regulatory activity of proteins could be affected by pH-dependent conformational changes, as shown in *L*. *lactis* GadR [38]. Thus it is conceivable that neutral pH is required to maintain the conformation of PmrA for DNA binding. However, the shift pattern in EMSA reactions at pH 5.5 was similar to that in reactions at pH 8 (data not shown), suggesting that acidic pH has limited effects on the conformation and/or DNA binding capacity of GlnR and PmrA. Furthermore, the intracellular pH of streptococcal cells grown at pH 5.5 generally is higher than the environmental pH value, thus the availability of the cofactor, rather than pH is likely to be the key control of the regulatory activity of GlnR and PmrA *in vivo*.

A closer examination of the predicted GlnR box in the promoters of all genes/operons of the GlnR regulon revealed that the putative GlnR box in p_*pmrA*_ bears an imperfect palindrome spaced by 5 bases (5’-TGTAG-N_5_-CTACA), rather than the 7-base in the proposed GlnR box consensus (5’-TGTNA-N_7_-TNACA). Since p_*pmrA*_ is the only promoter that showed a higher affinity for PmrA compared to GlnR based on the result of EMSA (Fig 3), variations in the binding consensus may exist for these two proteins. On the other hand, a pII homolog (SMUGS5_RS07465) is located 3’ to and co-transcribed with *nrgA* in *S*. *mutans* GS5. The PII proteins are the key regulators in the regulation of nitrogen metabolism in bacteria and archaea [39], and an interaction between the PII homolog and GlnR has also been demonstrated in *S*. *mutans* UA159 by *in vitro* analyses [40]. Thus, it is suggested that the activity of GlnR, and likely also PmrA, is modulated by the PII homolog. Whether the degradation of PmrA is prevented by NrgA and the PII homolog under limited nitrogen nutrients, as described in *B*. *subtilis* [24, 25], is currently under investigation.

Enhanced biofilm formation in strains ΔPmrA and ΔGlnR compared to wild-type GS5 is likely caused by downstream effects of the mutations since PmrA and GlnR regulate the GlnR regulon differently. However, it is unlikely that elevated biofilm formation in strains ΔGlnR and ΔPmrA was caused by enhanced glucan production via the activity of glucosyltransferases, as the biofilm assay was carried out in medium supplemented with glucose (BMG) but not sucrose. It remains possible that additional targets are regulated by GlnR and PmrA. Alternatively, GlnR and/or PmrA modulate biofilm formation indirectly. For instance, it is noticed that the chain length of strains ΔGlnR and ΔPmrA is more than twofold longer than that of wild-type GS5 in BMG. Although the nature of this phenotype is unknown, it is possible that the elongated chain length may enhance biofilm development. Additionally, in a mixed-strain biofilm study in which a mixture containing an equal amount of wild-type GS5 and strain ΔPmrA was inoculated into wells of a 96-well microtiter plate, we found that the viable count of wild-type GS5 in the biofilm was approximately 1.5-fold higher than that of strain ΔPmrA after 24-hour incubation, indicating that strain ΔPmrA competed less well than did wild-type GS5. Also in agreement with the result presented in Fig 6A, the presence of strain ΔPmrA in the mixed culture enhanced overall biofilm formation compared to cultures containing wild-type GS alone (data now shown). This observation suggests that inactivation of *pmrA* could enhance the colonization, but weaken the fitness of the bacteria within the biofilm. As a late colonizer of dental plaque, *S*. *mutans* seems to equip itself mainly with a strong capacity to cope with the alterations in the growth environment, rather than the ability to colonize the tooth surface.

## Conclusions

**Fig 7:**
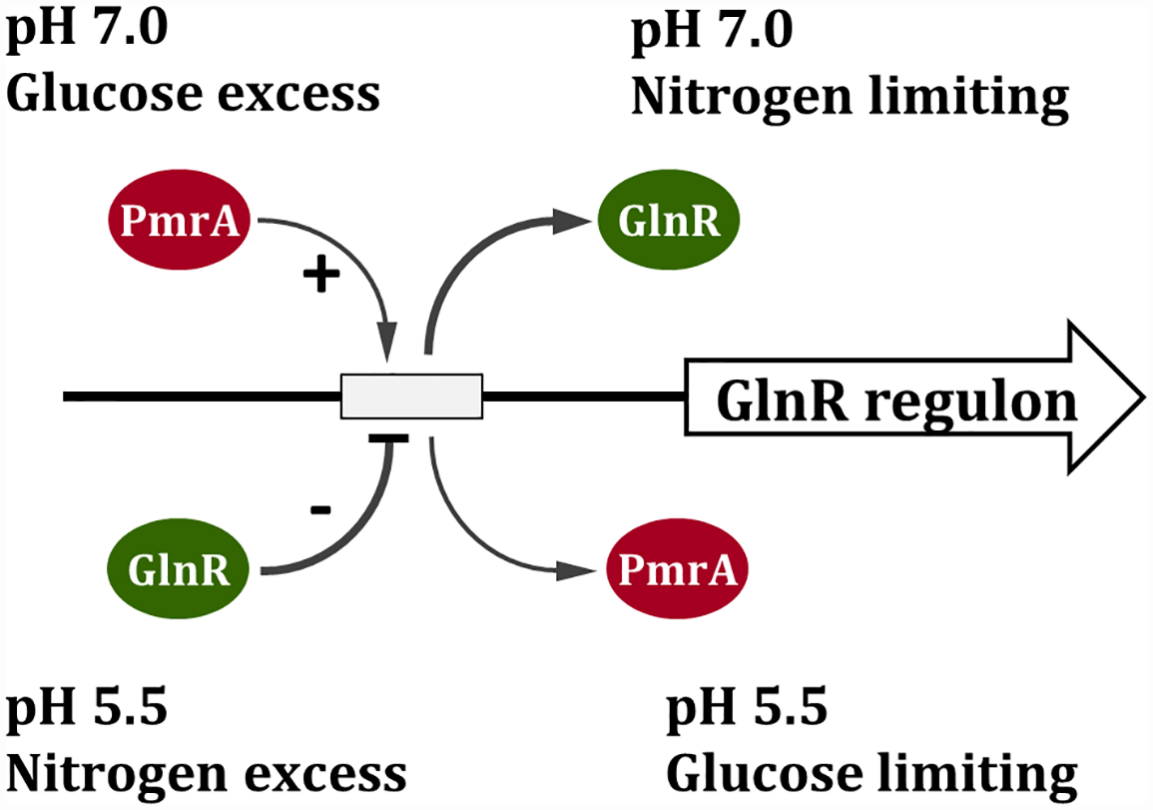
Model for the regulation of the GlnR regulon in *S*. *mutans* GS5. The putative GlnR box in the promoter is indicated by an open rectangle. The model suggests that PmrA binds to the GlnR box and activates the expression at pH 7, whereas GlnR represses the expression at acidic pH. Such regulation allows a quick switch between nitrogen metabolism and acid tolerance.

Taken together, a working model for the regulation of the GlnR regulon is proposed (Fig 7). At neutral pH, PmrA activates the expression of the GlnR regulon and the activation is augmented by glucose excess, allowing for optimal aa biosynthesis. At acidic pH and/or under nitrogen excess, GlnR represses the expression of the regulon for acid tolerance.

## Supporting Information

S1 Fig EMSA demonstrating the specific interaction of GlnR and PmrA to the GlnR box. The specific interaction of GlnR (A) and PmrA (B) to the GlnR box in p_*glnR*_, p_*glnQ*_, and p_*gdhA*_ are shown. All reactions were performed with 0.025 pmol of biotin-labeled probes. The reaction mixture was separated on a 6% polyacrylamide gel. Lane 1, reactions containing the wild-type probe only. Lanes 2 to 5, reactions containing the wild-type probe and 16 nM to 128 nM of MBP-GlnR (A) and MBP-PmrA (B) in twofold increments, respectively. Lane 6, reactions containing the mutated probe only. Lanes 7 to 10, reactions containing the mutated probe and 16 nM to 128 nM of MBP-GlnR (A) and MBP-PmrA (B) in twofold increments, respectively. (C) Sequences of the wild-type GlnR box and the mutated box in p_*glnR*_, p_*glnQ*_, and p_*gdhA*_ are shown. The mutated bases are in lowercase letters.
(PDF)

S2 Fig The impact of growth pH and carbohydrate concentrations on the expression of the GlnR regulon genes in *S*. *mutans* GS5. Wild-type GS5 was grown in chemostat in TY containing 20 mM (I) or 100 mM glucose (II) at pH 7.2 or pH 5.5. The relative quantity of mRNA of the GlnR regulon gene was measured by qPCR. The change in Δ*Cq* of each sample was normalized with 16S RNA. The Δ*Cq* of wild-type GS5 grown at pH 7.2 with 20 mM glucose was used as the reference. Numbers are the means and standard deviations of three independent experiments. The significant difference between samples under each growth condition was determined by Student’s *t* test. **, *P* < 0.001; *, *P* < 0.01, #, *P* < 0.05.
(PDF)

S3 Fig The expression of *dnaA*, *dnaE*, and *gyrA* is not regulated by PmrA. Total cellular RNA was isolated from wild-type GS5 and strain ΔPmrA grown in chemostat in TY containing 20 mM (A) or 100 mM glucose (B) at pH 7.2 or pH 5.5. The relative quantity of mRNA of *dnaA*, *dnaE*, and *gyrA* was measured by qPCR. The change in Δ*Cq* of each sample was normalized with 16S RNA. The Δ*Cq* derived from wild-type GS5 grown at pH 7.2 was used as the reference. Numbers are the mean and standard deviation of three samples.
(PDF)

S1 Table Primers used in this study. (PDF)
