# Supplementary material for: The GlnR Regulon in Streptococcus mutans Is Differentially Regulated by GlnR and PmrA
Source: PLoS One. 2016 Jul 25;11(7):e0159599. doi: 10.1371/journal.pone.0159599 (PMC4959772; doi:10.1371/journal.pone.0159599)
Supplement: S1 Fig — The specific interaction of GlnR (A) and PmrA (B) to the GlnR box in pglnR, pglnQ, and pgdhA are shown. All reactions were performed with 0.025 pmol of biotin-labeled probes. The reaction mixture was separated on a 6% polyacrylamide gel. Lane 1, reactions containing the wild-type probe only. Lanes 2 to 5, reactions containing the wild-type probe and 16 nM to 128 nM of MBP-GlnR (A) and MBP-PmrA (B) in twofold increments, respectively. Lane 6, reactions containing the mutated probe only. Lanes 7 to 10, reactions containing the mutated probe and 16 nM to 128 nM of MBP-GlnR (A) and MBP-PmrA (B) in twofold increments, respectively. (C) Sequences of the wild-type GlnR box and the mutated box in pglnR, pglnQ, and pgdhA are shown. The mutated bases are in lowercase letters. (PDF) [file pone.0159599.s001.pdf]

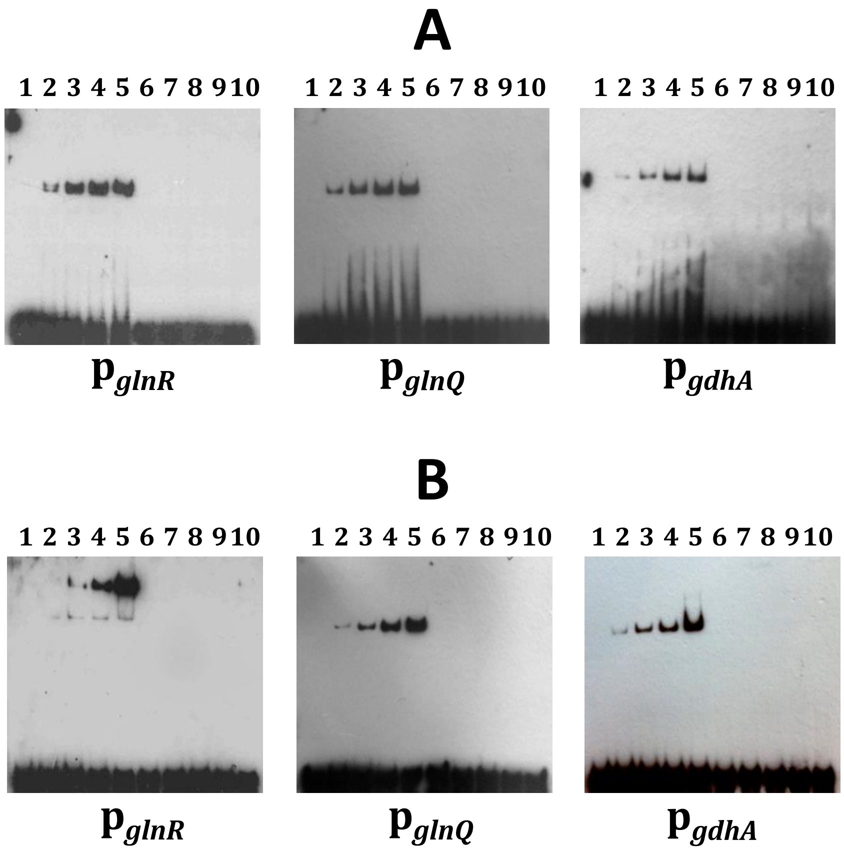

C

p_{glnR}	wild-type	5′-TGTCAGGAAATATGACA
	mutated	5′-ccattGGAAATActtct
p_{glnQ}	wild-type	5′-TGTTAGAAAAGCTAACA
	mutated	5′-ttgatGAAAAGCattag
p_{gdhA}	wild-type	5′-TGTTATTTTTTATAACA
	mutated	5′-caattTTTTTTAtctat

S1 Fig. EMSA demonstrating the specific interaction of GlnR (A) and PmrA (B) to the GlnR box in p_{glnR}, p_{glnQ}, and p_{gdhA}. All reactions were performed with 0.025 pmole of biotin-labeled probes. The reaction mixture was separated on a 6% polyacrylamide gel. Lane 1, reactions containing the wild-type probe only. Lanes 2 to 5, reactions containing the wild-type

probe and 16 nM to 128 nM of MBP-GlnR (A) and MBP-PmrA (B) in twofold increments, respectively. Lane 6, reactions containing the mutated probe only. Lanes 7 to 10, reactions containing the mutated probe and 16 nM to 128 nM of MBP-GlnR (A) and MBP-PmrA (B) in twofold increments, respectively. (C) Sequences of the wild-type GlnR box and the mutated box in p_{glnR}, p_{glnQ}, and p_{gdhA} are shown. The mutated bases are in lowercase letters.
