# Supplementary material for: The GlnR Regulon in Streptococcus mutans Is Differentially Regulated by GlnR and PmrA
Source: PLoS One. 2016 Jul 25;11(7):e0159599. doi: 10.1371/journal.pone.0159599 (PMC4959772; doi:10.1371/journal.pone.0159599)
Supplement: S2 Fig — Wild-type GS5 was grown in chemostat in TY containing 20 mM (I) or 100 mM glucose (II) at pH 7.2 or pH 5.5. The relative quantity of mRNA of the GlnR regulon gene was measured by qPCR. The change in ΔCq of each sample was normalized with 16S RNA. The ΔCq of wild-type GS5 grown at pH 7.2 with 20 mM glucose was used as the reference. Numbers are the means and standard deviations of three independent experiments. The significant difference between samples under each growth condition was determined by Student’s t test. **, P < 0.001; *, P < 0.01, #, P < 0.05. (PDF) [file pone.0159599.s002.pdf]

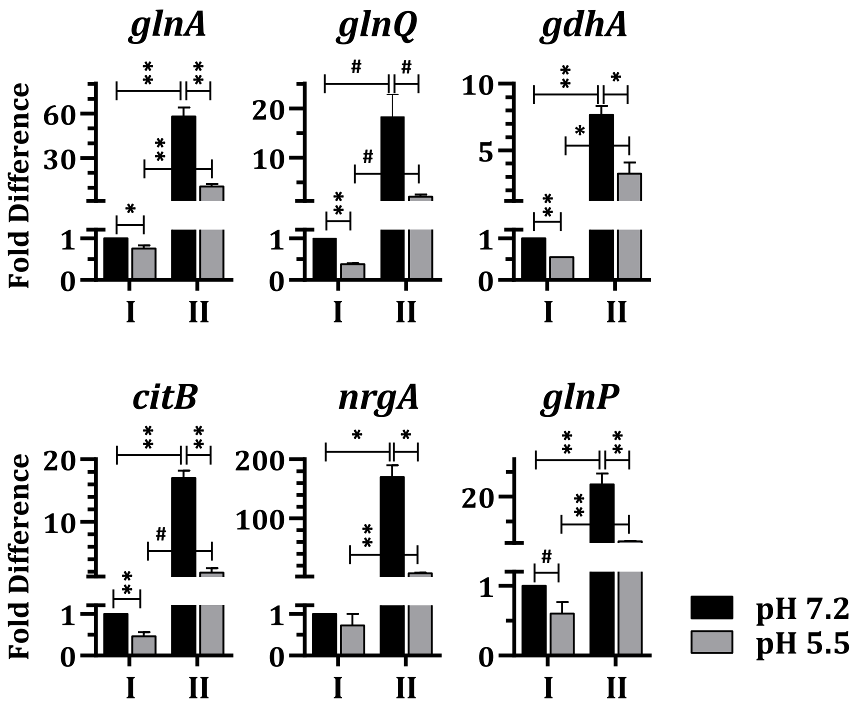

S2 Fig. The impact of growth pH and carbohydrate concentrations on the expression of the GlnR regulon genes in *S. mutans* GS5. Wild-type GS5 was grown in chemostat in TY containing 20 mM (I) or 100 mM glucose (II) at pH 7.2 or pH 5.5. The relative quantity of mRNA of the GlnR regulon gene was measured by qPCR. The change in Δ*Cq* of each sample was normalized with 16S RNA. The Δ*Cq* of wild-type GS5 grown at pH 7.2 with 20 mM glucose was used as the reference. Numbers are the means and standard deviations of three independent experiments. The significant difference between samples under each growth condition was determined by Student's *t* test. **, *P* < 0.001; *, *P* < 0.01, #, *P* < 0.05.
