# Supplementary material for: The GlnR Regulon in Streptococcus mutans Is Differentially Regulated by GlnR and PmrA
Source: PLoS One. 2016 Jul 25;11(7):e0159599. doi: 10.1371/journal.pone.0159599 (PMC4959772; doi:10.1371/journal.pone.0159599)
Supplement: S3 Fig — Total cellular RNA was isolated from wild-type GS5 and strain ΔPmrA grown in chemostat in TY containing 20 mM (A) or 100 mM glucose (B) at pH 7.2 or pH 5.5. The relative quantity of mRNA of dnaA, dnaE, and gyrA was measured by qPCR. The change in ΔCq of each sample was normalized with 16S RNA. The ΔCq derived from wild-type GS5 grown at pH 7.2 was used as the reference. Numbers are the mean and standard deviation of three samples. (PDF) [file pone.0159599.s003.pdf]

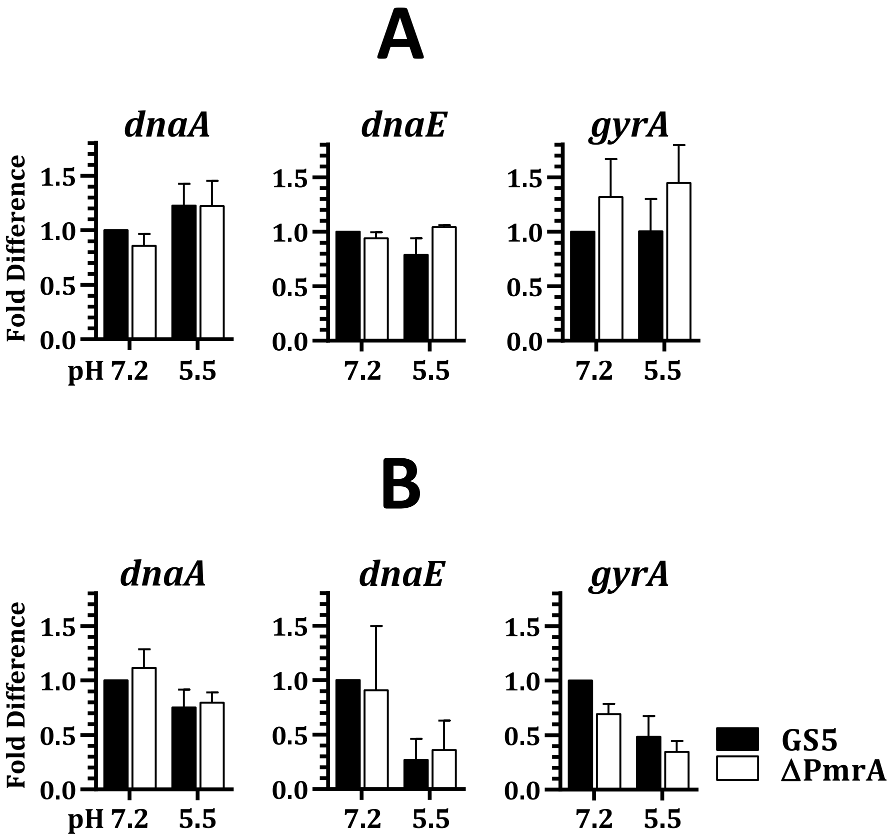

S3 Fig. The expression of *dnaA*, *dnaE*, and *gyrA* is not regulated by PmrA. Total cellular RNA was isolated from wild-type GS5 and strain ΔPmrA grown in chemostat in TY containing 20 mM (A) or 100 mM glucose (B) at pH 7.2 or pH 5.5. The relative quantity of mRNA of *dnaA*, *dnaE*, and *gyrA* was measured by qPCR. The change in ΔCq of each sample was normalized with 16S RNA. The ΔCq derived from wild-type GS5 grown at pH 7.2 was used as the reference. Numbers are the mean and standard deviation of three samples.
