# Supplementary material for: The GlnR Regulon in Streptococcus mutans Is Differentially Regulated by GlnR and PmrA
Source: PLoS One. 2016 Jul 25;11(7):e0159599. doi: 10.1371/journal.pone.0159599 (PMC4959772; doi:10.1371/journal.pone.0159599)
Supplement: S1 Table — (PDF) [file pone.0159599.s004.pdf]

S1 Table. Primers used in this study.

Primer	Sequence[a]	Purpose
glnRA_969F	GGGTGGAGGTGCAAGCATG	construction of ΔGlnR and GlnR+/ΔGlnR
glnRA_2033R	TTTCTCGAGTTTCCTCCTTTCATTGTC	construction of ΔGlnR
glnRA_2389F	TACGCATGCAAATACGTAACGTGGAATT	
glnRA_3403R	GGTGAACGATTGCGTCCCG	construction of ΔGlnR and GlnR+/ΔGlnR
glnR_C_1945R	GACGCATGCTAAACTTTACTTTTTGTAAAAAATTTTCG	construction of GlnR+/ΔGlnR
glnR_C_1946F	CTCCTGCAGGAGTTTATGTCAGGAAATATGACAT	
pmrA_3574F	CATGCCAATCACACCTAAAGC	construction of ΔPmrA and PmrA+/ΔPmrA
pmrA_4544R	AATGGATCCGAATAATCTTCGTTTCTT	construction of ΔPmrA
pmrA_4827F	GGACTCGAGGCCATTGAATTCTGAAGAC	
pmrA_5860R	CTGGCAGCAGGTTTTCAAGG	construction of ΔPmrA and PmrA+/ΔPmrA
pmrA_C_4860R	CTTGCATGCTTATTTGTCTTCAGAATTCAATGGCC	construction of PmrA+/ΔPmrA
pmrA_C_4861F	CACCTGCAGAAAAGAGTGGAACACTTTGGTTC	
qPCR_16s_F	TGGAATTCCATGTGTAGCGG	qPCR of 16s RNA
qPCR_16s_R	CCTGCCCGGGAACGTATTC	
qPCR_citB_F	TCAATTCTTACGGTAGCCGTCG	qPCR of *citB*
qPCR_citB_R	TTTACGCACAACCATTGG	
qPCR_gdhA_F	CTCTTACTGGCCAACCAATC	qPCR of *gdhA*
qPCR_gdhA_R	CACCACCTGCAGGTGTGAA	
qPCR_glnA_F	TGTTGAAATCCCAGCTAC	qPCR of *glnA*
qPCR_glnA_R	CAGTTGGATTCATGATAGC	
qPCR_glnP_F	CTGGCGATTCCATGTACAAC	qPCR of *glnP*
qPCR_glnP_R	CGACATTGCTTCCTTTAC	
qPCR_glnQ_F	CTACCATGCACTTCGTAA	qPCR of *glnQ*
qPCR_glnQ_R	AGAACTGTTGAGCTCGTG	
qPCR_nrgA_F	TCGCGATTATGACCGTC	qPCR of *nrgA*
qPCR_nrgA_R	CCCATAAAGGTTGGATAC	

MBP_glnR_F	GGGAATTCATGAAAGAAAAAGAGCTTCGAC	construction of MBP-GlnR
MBP_glnR_R	GTTCTGCAGTTACATACGTAAACCACCAAGA	
MBP_pmrA_F	CTGGATCCATGGAAAAAAGAAACGAAGATT	construction of MBP-PmrA
MBP_pmrA_R	CACCTGCAGTTATTTGTCTTCAGAATTCAAT	
EMSA_citB_F	CTATTTTTATGTTATAAAACATAACATAAAATGCAAGCTT	EMSA probes
EMSA_citB_R	AAGCTTGCATTTTATGTTATGTTTTATAACATAAAAATAG	
EMSA_gdhA_F	TTAATCTAATGAATGTTATTTTTTATAACATGAAAGAATA	
EMSA_gdhA_R	TATTCTTTCATGTTATAAAAAATAACATTCATTAGATTAA	
EMSA_glnP_F	TATAGACTGTTATGTAAGGAAATATAACATCTATTATTAA	
EMSA_glnP_R	TTAATAATAGATGTTATATTTCCTTACATAACAGTCTATA	
EMSA_glnQ_F	GTATAACAATATAATGTTAGAAAAGCTAACAATAAATTTA	
EMSA_glnQ_R	TAAATTTATTGTTAGCTTTTCTAACATTATATTGTTATAC	
EMSA_glnR_F	GTTTAGAGTTTATGTCAGGAAATATGACATATAGGCTTGA	
EMSA_glnR_R	TCAAGCCTATATGTCATATTTCCTGACATAAACTCTAAAC	
EMSA_nrgA-1_F	TACCTCCCTTTATGTTATATAATATAACATTATTCCTAAA	
EMSA_nrgA-1_R	TTTAGGAATAATGTTATATTATATAACATAAAGGGAGGTA	
EMSA_nrgA-2_F	CTTTTTTGTTATATGTTATGTTTTTTAACATTTCTATCTA	
EMSA_nrgA-2_R	TAGATAGAAATGTTAAAAAACATAACATATAACAAAAAA G	
EMSA_pmrA_F	TAAACATTTTATGTAGAAAAACTACATTTATTGTAATATA	
EMSA_pmrA_R	TATATTACAATAAATGTAGTTTTTCTACATAAAATGTTTA	
EMSA_gdhA_Con_F	TTAATCTAATGAAcaattTTTTTTAtctatTGAAAGAATA	EMSA control probes with mutated bases in the GlnR box
EMSA_gdhA_Con_R	TATTCTTTCAatagaTAAAAAAaattgTTCATTAGATTAA	
EMSA_glnQ_Con_F	GTATAACAATATAAttgatGAAAAGCattagATAAATTTA	
EMSA_glnQ_Con_R	TAAATTTATctaatGCTTTTCatcaaTTATATTGTTATAC	
EMSA_glnR_Con_F	GTTTAGAGTTTAccattGGAAATActtctTATAGGCTTGA	
EMSA_glnR_Con_R	TCAAGCCTATAagaagTATTTCCaatggTAAACTCTAAAC	

[a] Inserted restriction sites are underlined. The mutated bases in the GlnR box are in lower case.

CTCGAG, the XhoI recognition sequence; GCATGC, the SphI recognition sequence; CTGCAG, the PstI recognition sequence; GGATCC, the BamHI recognition sequence; GAATTC, the EcoRI recognition sequence.
